# The Roles of Hypoxia-Inducible Factors and Non-Coding RNAs in Gastrointestinal Cancer

**DOI:** 10.3390/genes10121008

**Published:** 2019-12-04

**Authors:** Hyun-Soo Cho, Tae-Su Han, Keun Hur, Hyun Seung Ban

**Affiliations:** 1 Korea Research Institute of Bioscience and Biotechnology, Daejeon 34141, Korea; chohs@kribb.re.kr (H.-S.C.);; 2 Department of Biochemistry and Cell Biology, School of Medicine, Kyungpook National University, Daegu 41944, Korea

**Keywords:** hypoxia, non-coding RNA, microRNA, hypoxia-inducible factor, gastrointestinal cancer

## Abstract

Hypoxia-inducible factors (HIFs) are transcription factors that play central roles in cellular responses against hypoxia. In most cancers, HIFs are closely associated with tumorigenesis by regulating cell survival, angiogenesis, metastasis, and adaptation to the hypoxic tumor microenvironment. Recently, non-coding RNAs (ncRNAs) have been reported to play critical roles in the hypoxic response in various cancers. Here, we review the roles of hypoxia-response ncRNAs in gastrointestinal cancer, with a particular focus on microRNAs and long ncRNAs, and discuss the functional relationships and regulatory mechanisms between HIFs and ncRNAs.

## 1. Introduction

Gastrointestinal (GI) cancer is the most common type of cancer and second leading cause of cancer-related death [1]. GI cancer is particularly difficult to treat since it is usually found in an advanced stage. The abnormal growth of cancer cells induces hypoxic conditions in most solid tumors as well as GI cancers [2]. Hypoxia-inducible factors (HIFs) are heterodimeric transcription factors consisting of two oxygen response subunits (HIF-1α and -2α) and a constitutively expressed subunit (aryl hydrocarbon receptor nuclear translocator; ARNT, also known as HIF-1β) [3,4]. In the presence of oxygen, the posttranslational proline hydroxylation of HIF-1α by prolyl hydroxylase (PHD) leads to E3 ubiquitin ligase von Hippel-Lindau (VHL)-mediated ubiquitination, resulting in HIF-1α degradation via the ubiquitin-proteasome system [5]. Under hypoxic conditions, HIF-1α translocates into the nucleus where it dimerizes with HIF-1β and binds to hypoxia-response elements (HREs; 5′-RCGTG-3′, where R is A or G) in the promoter regions of target genes, including erythropoietin (EPO), vascular endothelial growth factor (VEGF), glucose transporters, and glycolytic enzymes [6,7,8]. HIFs are therefore pivotal regulators of the tumor microenvironment and are involved in cancer cell growth, migration, angiogenesis, and resistance to cancer therapy [9,10]. Indeed, enhanced HIF-1α expression has been detected in various cancers, including lung, breast, colon, and liver cancers, and is significantly correlated with low patient survival rates [9,11]. Thus, HIFs are an attractive therapeutic target for treating cancer.

MicroRNAs (miRNAs) are small non-coding RNAs (ncRNAs) whose first transcripts (pri-miRNAs) are typically produced by RNA polymerase II [12]. Pri-miRNAs are then cleaved by a microprocessor complex (DGCR8 and Drohsa) into stem-loop structures of ~70–110 nucleotides (nt), known as pre-miRNAs. These pre-miRNAs are translocated from the nucleus to the cytoplasm by the Ran/GTP/Exportin5 complex, where they are then processed into mature duplex miRNAs of 21–25 nt by the RNase III enzyme, Dicer. After the duplex has been unwound, mature miRNAs can regulate their target genes alongside the RNA-induced silencing complex (RISC) [13,14], playing important roles in post-transcriptional regulation. The first miRNA to be identified was lin-4 in *Caenorhabditis elegans* (*C. elegans*) [15], which affects development by regulating the lin-14 protein. After seven years, another miRNA was reported in *C. elegans*, let-7, which negatively regulates lin-41 via sequence-specific interactions with its 3′-untranslated region (3′-UTR) [16]. The development of microarray and RNA sequencing techniques have led to the discovery of numerous novel and conserved miRNAs in vertebrates. We currently know of over 1800 annotated human precursor miRNAs that are processed into over 2500 mature miRNA sequences; however, the functions of many of these miRNAs in various aspects of cancer remain unknown.

Long non-coding RNAs (lncRNAs), which are generally over 200 nt in length (typically 1000–10000 nt) and have no protein-coding potential, are now known to be important regulators of cellular processes such as development and metabolism. LncRNAs are transcribed by RNA polymerase II and undergo multiple exon splicing [17,18]; however, their dysregulation is widely associated with diseases such as cancer [19], with abnormal lncRNA transcript expression linked to the progression and metastasis of cancer and patient survival. LncRNAs can also act as tumor suppressors (e.g. BGL3, GAS5, PTENP1, and MEG3) or oncogenes (e.g. HOTAIR, MALAT1, PCAT1/5/18, PVT1, UCA1, and XIST) in cancer [20,21,22], and can act as sponges to regulate miRNAs by competitively binding with their target genes, thereby increasing miRNA target gene expression. However, despite the close link between lncRNAs and cancer progression, their functions in cancer are not fully understood.

Here, we review the close relationship between HIFs and ncRNAs, the regulation of their expression, molecular function, and cellular responses to hypoxia in GI cancers. Furthermore, we discuss the potential therapeutic applications of ncRNAs to treat hypoxic cancers.

## 2. HIF-Related ncRNAs in Gastric Cancer (GC)

### 2.1. ncRNAs Regulate HIF Expression in GC

Many studies have demonstrated that altered miRNA expression is involved in hypoxia in cancer by targeting HIFs. For instance, miR-18a expression is downregulated under hypoxic conditions in GC cell lines [23], with miR-18a overexpression increasing the number of apoptotic cells and decreasing cell invasion by directly targeting HIF-1α. In addition, miR-186 has been reported to have anti-proliferative roles in some cancers; in GC, miR-186 suppresses cell proliferation by negatively regulating HIF-1α [24] and consequently affects downstream HIF-1α target genes, including PD-L1, hexokinase 2, and PFKP. Therefore, miR-186 functions as a tumor suppressor in GC. HIF-1α expression is also negatively regulated by miR-143-5p, but is positively correlated with the expression of the lncRNA ZEB2-AS1 in GC [25]. Bioinformatic analysis has revealed that ZEB2-AS1 can be regulated by miR-143-5p; therefore, ZEB2-AS1-mediated GC progression may involve the miR-143-5p/HIF-1α axis.

It has also been shown that overexpression of the lncRNA plasmacytoma variant translocation1 (PVT1) promotes multidrug resistance in GC cells [26], with high PVT1 expression observed in cisplatin-resistant GC patients. PVT1 knockdown has been found to reduce cisplatin resistance and increase apoptosis in GC cells, while PVT1 overexpression has anti-apoptotic effects and increases the expression of the drug resistance-related genes MDR1, MRP, mTOR, and HIF-1α. Other studies have shown that PVT1 can regulate miR-186 [27] and that PVT1 expression is upregulated in GC tissues and associated with lymph node metastasis. Furthermore, a functional study found that PVT1 overexpression promotes cell proliferation and invasion, with PVT1 upregulation increasing HIF-1α levels by sponging miR-186 in GC.

Crocin is a key bioactive compound with well known anti-inflammatory, anti-oxidant, and anti-cancer effects. In GC, crocin has been found to suppress the migration, invasion, and epithelial-to-mesenchymal transition (EMT) of GC cells by modulating miR-320/KLF5/HIF-1α signaling [28]. Analysis of clinical GC samples revealed that both KLF5 and HIF-1α expression are upregulated in GC tissues and that KLF5 expression is positively correlated with HIF-1α expression. Moreover, crocin treatment decreases KLF5 and HIF-1α expression but increases miR-320 expression, with target gene validation revealing that miR-320 negatively regulates KLF5 expression in GC. Although miR-320 regulates HIF-1α indirectly, its signaling is important for understanding the regulatory mechanism of HIF-1α.

### 2.2. HIFs Regulate ncRNA Expression in GC

miR-210 is a representative hypoxia-specific miRNA that has been identified in various tumor types, including GC, with hypoxic conditions able to induce miR-210 expression in SGC-7901 GC cells in a time dependent manner [29]. By acting as a transcription factor, HIF-1α can transcriptionally activate and upregulate miR-210 expression, which can affect chemoresistance, invasion, and metastasis by inhibiting its target gene, HOXA9, and increasing the expression of the EMT-related genes vimentin and N-cadherin. Moreover, Chen et al. found that miR-210 expression is regulated by epigenetic factors, with oxLDL decreasing DNA methylation at the miR-210 promoter site where HIF-1α binds [30]. This hypomethylation of the miR-210 promoter by oxLDL increases the likelihood of HIF-1α binding to the miR-210 promoter site in GC to upregulate miR-210 expression. Recently, Zhang et al. suggested that phosphatase of regenerating liver-3 (PRL-3) regulates GC cell invasiveness by acting as an upstream regulator of HIF-1α; PRL-3 activates NF-kB signaling and promotes HIF-1α expression, consequently inducing miR-210 expression [31]. These findings suggest that the PRL-3/NF-kB/miR-210 axis promotes cell migration and invasion, and is related to poor prognosis in patients with GC.

It has been reported that angiogenic miR-382 is also upregulated by hypoxia. Seok et al. identified miR-382 using a miRNA microarray technique with MKN1 human GC cells, finding that miR-382 is upregulated by HIF-1α under hypoxic conditions [32]. Moreover, miR-382 upregulation decreases the expression of its target genes, phosphatase, and tensin homolog (PTEN) and activates the AKT/mTOR signaling pathway, indicating that HIF-1α-induced miR-382 promotes angiogenesis and acts as an oncogene by targeting PTEN in GC. The same research group also analyzed the clinical significance and prognostic role of hypoxia-induced miR-382 in GC [33], finding that miR-382 is significantly upregulated in GC tissues and could therefore be a prognostic marker for advanced GC. These reports suggest that miR-382 plays important roles in malignant GC development. MiR-107 has also been studied as a hypoxia-specific miRNA in GC. Analysis of GC patient serum and tissue samples found that miR-107 expression is higher in both sample types and positively correlated with HIF-1α expression [34], suggesting that miR-107 could be used as a diagnostic biomarker for patients with GC.

HIF-1α-mediated miRNA expression has also been found to affect drug resistance. HIF-1α knockdown decreases miR-27a levels and inhibits the proliferation of GC cells, with dual luciferase reporter assays and ChIP analysis revealing that HIF-1α can directly bind to the miR-27a promoter and enhance its transcriptional activity [35]. Moreover, suppressing HIF-1α or miR-27a decreased MDR1/P-gp, Bcl-2, and LRP expression, suggesting that HIF-1α is closely associated with multi-drug resistance in GC via miR-27a expression. miR-421 is also a drug resistance-related miRNA induced by HIF-1α in GC [36], whose upregulated expression in GC tissues is associated with decreased patient survival. Overexpressing miR-421 in GC cells was found to promote metastasis and induce cisplatin resistance in vivo and in vitro, exerting its oncogenic functions by targeting the E-cadherin and caspase-3 genes.

Other miRNAs, such as miR-214 and miR-224, have also been identified as hypoxia-inducible miRNAs in GC [37,38]. Both miR-214 and miR-224 are upregulated in GC tissue samples, with their overexpression promoting the growth and migration of GC cells and their inhibition having the opposite effects under hypoxic conditions. Furthermore, target gene validation revealed that miR-224 directly regulates RASFF8, which reduces the transcriptional activity of NF-kB and p65 translocation, whereas miR-214 negatively regulates the adenosine A2A receptor (A2AR) and PR/SET domain 16 (PRDM16) in GC.

It has been reported that the lncRNA, urothelial cancer associated 1 (UCA1) is upregulated in hypoxia-resistant GC cell lines and promotes their migration [39]. Bioinformatics analysis and in vitro assays revealed that miR-7-5p can bind to UCA1-specific sites to regulate EGFR by reducing the binding efficiency of miR-7-5p for the EGFR transcripts. Therefore, hypoxia-induced UCA1 promotes cell migration by increasing EGFR expression in GC. Hypoxia can also induce the lncRNA BC005927 in GC cells, which is involved in hypoxia-induced metastasis [40], frequently upregulated in GC samples, and associated with poor prognosis, with high BC005927 expression decreasing the overall survival of patients with GC. ChIP and luciferase reporter assays have shown that BC005927 is directly regulated by HIF-1α and upregulated by the metastasis-related gene, EPHB4. Therefore, hypoxic conditions induce HIF-1α, BC005927, and EPHB4 expression in GC cells, resulting in increased metastasis. Another hypoxia-induced lncRNA, prostate cancer gene expression marker 1 (PCGEM1), has also been identified in GC [41]. PCGEM1 is also overexpressed in GC tissues and its expression is upregulated by hypoxia in GC cells, with PCGEM1 knockdown significantly repressing GC cell invasion and metastasis. Additionally, PCGEM1 positively regulates the expression of SNAI1, a key transcription factor for EMT. Gastric adenocarcinoma associated, positive CD44 regulator, long intergenic non-coding RNA (GAPLINC) is also upregulated in GC tissues and associated with poor prognosis; moreover, it is directly activated by HIF-1α at the transcriptional level in GC, with GAPLINC knockdown suppressing hypoxia-induced tumor growth in vivo [42].

Microarray techniques have been used to identify lncRNAs that are differentially expressed under hypoxic conditions, including AK123072 and AK058003 which are upregulated by hypoxia [43,44]. Both are frequently upregulated in GC samples and promote cell migration and invasion, with AK123072 positively correlated with EGFR expression and AK058003 positively correlated with γ-synuclein (SNCG) expression in GC samples. Thus, these techniques may help develop therapeutic agents against GC. HIF-related ncRNAs in GC are summarized in Table 1.

## 3. HIF-Related ncRNAs in Colorectal Cancer (CRC)

### 3.1. ncRNAs Regulate HIF Expression in CRC

Circulating upregulated miR-210 and miR-21 and downregulated miR-126 expression have shown potential as diagnostic biomarkers for CRC as they are involved in the HIF-1α/VEGF signaling pathways for colon cancer initiation [45]. During EMT and mesenchymal-to-epithelial transition (MET), HIF-1α up-regulates the expression of Achaete scute-like2 (Ascl2), a transcriptional regulator of miR-200b, by binding to the HRE site at the Ascl2 promoter. Under hypoxic conditions, Ascl2 overexpression by HIF-1α induces EMT by repressing miR-200b; however, since HIF-1α is a direct target of miR-200b, the HIF-1α-Ascl2-miR-200b axis allows regulatory feedback for CRC EMT-MET plasticity [46]. In addition, miR-199a downregulation has been associated with CRC metastasis and incidence, while miR-199a overexpression suppresses the proliferation, migration, and invasion of CRC cell lines by reducing HIF-1α/VEGF expression [47].

During CRC development, factor inhibiting HIF-1α (FIH-1) represses the HIF-1α pathway [48], suggesting that the association between FIH and HIF affects tumor development. FIH-1 is a direct target of miR-31, which is overexpressed in CRC and associated with CRC development by reducing FIH expression. Treatment with miR-31 inhibitors has been shown to reduce cell growth, migration, and invasion by inducing FIH expression and reducing HIF-1α pathway signaling. In addition, in clinical CRC cohorts, miR-31 and FIH expression are negatively correlated [49], with miR-22 directly regulating HIF-1α expression by binding the 3’ UTR of HIF-1α. Furthermore, overexpressing miR-22 in HCT116 cell lines reduces VEGF expression and represses cell growth and invasion by downregulating HIF-1α expression [50].

In colon cancer, p53 transcriptionally regulates miR-107 to regulate hypoxic signaling, while miR-107 directly regulates HIF-1β expression. Overexpressing miR-107 negates the effects of hypoxia by reducing HIF-1β expression, whereas miR-107 knockdown induces hypoxic signaling by increasing HIF-1β expression. In vivo phenotype analysis found that miR-107 overexpression reduces tumor growth, VEGF expression, and angiogenesis in mice. Furthermore, a CRC cohort study found that miR-107 expression is inversely associated with HIF-1β expression [51].

In CRC cell lines, miR-145 expression is reduced and can directly regulate p70S6K1 expression by binding its 3’-UTR. Since HIF1-α and VEGF are downstream targets of p70S6K1, miR-145 overexpression can suppress CRC growth and angiogenesis by decreasing HIF-1α and VEGF expression. Correlation analysis revealed that miR-145 expression is negatively correlated with p70S6K1, suggesting that miR-145 acts as a tumor suppressor in CRC [52]. Under hypoxic conditions, autophagy is induced and is related to CRC metastasis and EMT. Expression of the lncRNA CPS1-IT1 is significantly reduced in CRC tissues and cell lines, with in vitro analysis revealing that CPS1-IT1 overexpression suppresses EMT and autophagy by inhibiting HIF-1α activation. The regulation of CRC metastasis by autophagy under hypoxic conditions may therefore be associated with CPS1-IT1 acting as a tumor suppressor [53].

### 3.2. HIFs Regulate ncRNA Expression in CRC

Under hypoxic conditions, tumor cells modify their energy sources to maintain malignant proliferation [8]. MiR-23a, miR-27a, and miR-24 are significantly overexpressed in CRC due to direct regulation by HIF-1α, which binds the HRE1 and HRE2 sites of the miR-23a~27a~24 cluster under hypoxic conditions. HIF-1α induction of the miR-23a~27a~24 cluster is critically associated with the glucose metabolic pathway as it controls TCA-related genes and metabolic pathways in CRC cell lines, suggesting that this mechanism may be an important metabolic switch in CRC metabolism [54].

In cancer, hypoxia-induced autophagy can lead to radioresistance [55]. Under hypoxic conditions, HIF-1α overexpression positively regulates miR-210 expression, which induces autophagy by inhibiting Bcl-2 expression, thus reducing radiosensitivity in CRC [56]. MiRNAs are strongly involved in the acquisition of drug resistance in CRC [57]. Under hypoxic conditions, HIF-1α mediates miR-338-5p downregulation, which is associated with hypoxia-induced drug resistance by regulating its direct target, IL-6. Furthermore, miR-338-5p overexpression and HIF-1α inhibitors have been shown to reduce CRC drug resistance to oxaliplatin in vivo [58].

Expression of the lncRNA HOTAIR is directly regulated by HIF-1α binding to its HRE site, while MLL1 (a histone methyltransferase) and p300 (a histone acetyltransferase) are epigenetically enriched at the HOTAIR promoter under hypoxic conditions, suggesting that both MLL1 and p300 coordination, as well as HIF-1α, are involved in the regulation of HOTAIR under hypoxia in CRC [59]. MiR-15-16 expression is regulated by hypoxia-induced repression in CRC. In advanced stage tumors, HIF-2α induces c-Myc to repress miR-15-16, which promotes tumor metastasis and angiogenesis by downregulating FGF2, a direct target of miR-15-16 [60]. There are no more literatures about HIF-2α-related ncRNA in CRC as well as GC, but a few studies have been reported to a lesser extent than HIF-1α in other cancers. For instance, miR-145 suppresses HIF-2α expression through targeting 3’-UTR of HIF-2α in neuroblastoma [61], and induction of NRAT1 lncRNA expression by HIF-2α leads to increase in cell proliferation in hypoxic breast cancer [62]. HIF-related ncRNAs in CRC are summarized in Table 2.

## 4. Prospective Therapeutic Applications of ncRNAs as HIF Regulators

It has been well established that HIFs exert tumorigenic effects in the tumor microenvironment, including regulating cell metabolism, proliferation, invasion, metastasis, angiogenesis, and resistance to chemo- and radiotherapy [9,63,64]. Therefore, HIFs are an attractive target for anti-cancer drugs that act specifically on hypoxic cancer cells. Considerable effort has been put into developing HIF regulators, with several inhibitors having entered clinical trials to treat a variety of cancers [65].

The HIF-1α inhibitor digoxin, which exerts anti-cancer effects by reducing the protein levels of HIF-1α and its target genes (GLUT1, HK, and VEGF [66]) is currently in phase II clinical trials to treat several cancers, including head and neck cancer, Kaposi’s sarcoma, and breast cancer [67]. The first-in-class HIF-2α inhibitors PT2385 and PT2977 reduce the hypoxia-induced expression of VEGF and other hypoxia-responsible genes by blocking HIF-2α dimerization and DNA binding [68,69]. These inhibitors are currently under evaluation in a phase I/II clinical trial for treating VHL-associated renal carcinoma (RCC) and advanced clear cell RCC [70,71].

Although small molecules have been widely developed as HIF regulators, many recent studies have proposed the use of miRNA-based approaches for regulating HIFs [72]. The detailed mechanisms via which miRNAs control HIF-1α expression can be divided into direct and indirect regulation. Most miRNAs, including miR-18a, miR-186, miR-143, miR-200b, and miR-22, suppress HIF-1α expression in GC by binding directly to its 3’-UTR in an oxygen-independent manner. Conversely, the indirect regulation of HIF-1α protein levels by miRNAs is an oxygen-dependent process. For instance, miR-182 reduces PHD expression by targeting its 3’-UTR, thus stabilizing HIF-1α proteins in prostate cancer [73]. FIH-1 is another miRNA that indirectly regulates HIF-1α; in response to oxygen, FIH-1 hydroxylates Asp803 in the HIF-1α C-terminal transcription activation domain, blocking the recruitment of its co-activator p300 and inactivating its transcriptional activity. Enhanced miR-31 expression reduces FIH-1 expression by binding its 3’-UTR and inducing HIF-1α activation [49] in head and neck carcinoma [74]. Therefore, both the direct and indirect mechanisms of HIF regulation are potential therapeutic targets for treating cancer.

It is well known that activating HIF signaling results in resistance to chemo- and radiotherapy in hypoxic cancer cells [75]. As mentioned previously, HIF-1α suppresses Bcl-2 expression in colon cancer cells by inducing miR-210 expression, resulting in autophagy activation and radioresistance [56]. MiR-210-induced radioresistance has also been reported in other cancer cells under hypoxic conditions, such as hepatocellular cancer cells [76] and glioma stem cells [77]. In addition, chemotherapy resistance is also associated with miRNA expression levels. In colorectal cancer, enhancing miR-31 expression using 5-FU, one of the most common anticancer drugs, can promote chemoresistance [78]. Since miR-31 directly suppresses FIH-1 expression [49], these studies suggest that HIF-1α is strongly involved in miR-31-mediated resistance. Furthermore, miR-31-specific oligonucleotides increase the 5-FU sensitivity of HCT116 colon cancer cells [79]. Therefore, miR-210 and miR-31 might both be attractive therapeutic targets to increase the sensitivity of hypoxic cancer cells to radiotherapy and chemotherapy.

Cancer immunotherapies that target immune checkpoints have been identified as promising cancer treatments; for instance, immune checkpoint-blocking monoclonal antibodies such as cytotoxic T-lymphocyte associated antigen (CTLA)-4 and programed death (PD)-1 have been approved by the Food and Drug Administration (FDA) to treat various cancers [80,81]. Under hypoxic conditions, HIF-1α enhances the expression of PD-1 ligand (PD-L1) on the surface of cancer cells by directly binding to the HRE in the PD-L1 promoter [82,83]. In addition, HIF-2α induces PD-L1 expression in VHL-deficient clear cell RCC cells [84,85], indicating that HIF-1α and -2α inhibition could sensitize cells to anti-PD-L1 therapy by downregulating PD-L1. Based on these findings, miRNAs that reduce HIF expression, such as miR-18a, miR-186, miR-143, miR-210, miR-21, miR-22, and miR-145, may also overcome the PD-L1-mediated immune escape of cancer cells in gastrointestinal cancers. Furthermore, miRNAs such as miR-570, miR-513, miR-197, miR-34a, and miR-200 have been found to negatively regulate PD-L1 expression at the post-transcriptional level by binding its 3′-UTR [86,87]. Thus, combining these miRNAs with HIF inhibitors could provide prospective PD-1/PD-L1-based cancer immunotherapies.

## 5. Summary

**Figure 1:**
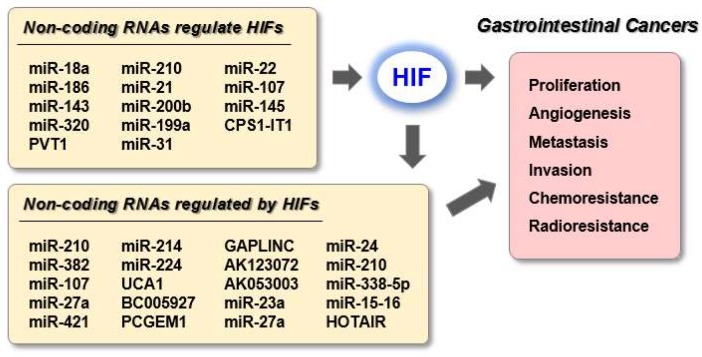
Summary for HIF-related ncRNAs in gastrointestinal cancers.

Here, we reviewed the roles of HIFs and ncRNAs in GI cancers. Various ncRNAs are involved in hypoxia-mediated cellular processes, including the regulation of HIF-1α expression, proliferation, angiogenesis, metastasis, and chemoresistance; thus, may be useful therapeutic targets or diagnostic biomarkers (Figure 1). Recently, there has been growing concern about cancer metabolism drugs that target the metabolic processes of cancer cells, such as aerobic glycolysis, mitochondrial metabolism, and energy utilization. Since HIFs play a central role in hypoxia-specific cancer metabolism, ncRNAs that downregulate HIF signaling combined with HIF inhibitors could be considered as novel cancer metabolic therapy strategies.

## Figures and Tables

**Table 1:** Hypoxia-inducible factor (HIF)-related non-coding RNAs (ncRNAs) in gastric cancer (GC).

Non-coding RNAs	Functions	Targets	Ref.
miR-18a	Promotes apoptosis and reduces cell invasion by regulating HIF-1α	HIF-1α	[23]
miR-186	Directly regulates HIF-1α and inhibits cell proliferation	HIF-1α	[24]
miR-143	Directly regulates HIF-1α	HIF-1α, ZEB2-AS1	[25]
PVT1	Enhances multidrug resistance and is associated with lymph node metastasis	HIF-1α, MDR1, MRP, mTOR	[26,27]
miR-320	Upregulated by crocin and regulates HIF-1α by inhibiting KLF5	KLF5	[28]
miR-210	Regulates chemoresistance, invasion, and metastasis (HRE region in miR-210 promoter ^1^)	HOXA9	[29,30]
miR-382	Enhances angiogenesis (HRE region in miR-382 promoter ^1^)	PTEN	[32,33]
miR-107	Upregulated in GC serum and tissue samples		[34]
miR-27a	Regulates cell proliferation and drug resistance (HRE region in miR-27a promoter ^1^)		[35]
miR-421	Upregulated in GC tissues. Regulates metastasis and cisplatin resistance (HRE region in miR-421 promoter ^1^)	CDH1, CASP3	[36]
miR-214	Upregulated in GC tissues. Enhances cell proliferation and migration	A2AR, PRDM16	[37]
miR-224	Regulates cell proliferation and migration (HRE region in miR-224 promoter ^1^)	RASFF8	[38]
UCA1	Promotes hypoxia-induced cell migration	miR-7-5p	[39]
BC005927	Upregulated in GC and is associated with poor prognosis. Promotes metastasis (HRE region in lncRNA promoter ^1^)	EPHB4	[40]
PCGEM1	Regulates GC cell invasion, metastasis, and EMT	SNAI1	[41]
GAPLINC	Upregulated in GC and is associated with poor prognosis (HRE region in lncRNA promoter ^1^)		[42]
AK123072	Upregulated in GC tissues. Regulates cell migration and invasion		[43]
AK053003	Regulates GC cell migration and invasion	SNCG	[44]

^1^ Mechanism of regulation by HIFs.

**Table 2:** HIF-related ncRNAs in colorectal cancer (CRC).

Non-coding RNAs	Functions	Targets	Ref.
miR-210, miR-21	Regulates HIF-1α/ VEGF signaling pathways to initiate colon cancer.		[45]
miR-200b	Directly regulates HIF-1α and regulatory feedback for CRC EMT-MET plasticity.	HIF-1α	[46]
miR-199a	Reduces HIF-1α/VEGF expression and suppresses CRC proliferation, migration, and invasion.		[47]
miR-31	Reduces FIH expression and is associated with CRC development.	FIH-1	[49]
miR-22	Down-regulates HIF-1α and represses cell growth and invasion in CRC.	HIF-1α	[50]
miR-107	Reduces HIF-1β expression and down-regulates tumor growth, VEGF expression, and angiogenesis.	HIF-1β	[51]
miR-145	Down-regulates HIF-1α and VEGF expression and represses cell growth and angiogenesis in CRC.	p70S6K1	[52]
lncRNA CPS1-IT1	Inhibits HIF-1α activation and suppresses EMT and autophagy.		[53]
miR-23a, miR27a, miR-24	Regulates glucose association via TCA-related genes under hypoxic conditions (HRE1, HRE2 in miRNA promoter ^1^).		[54]
miR-210	Reduces radiosensitivity by inducing autophagy in CRC.	Bcl-2	[56]
miR-338-5p	Reduces hypoxia-induced drug resistance by regulating IL-6.	IL-6	[58]
lncRNA HOTAIR	Regulates tumor growth under hypoxic conditions in CRC (HRE region in lncRNA promoter ^1^).		[59]
miR-15-16	Regulates tumor metastasis and angiogenesis (Indirect target of HIF-2α ^1^).	FGF-2	[60]

^1^ Mechanism of regulation by HIFs
